# Patients with persistent idiopathic dentoalveolar pain in dental practice

**DOI:** 10.1111/iej.13664

**Published:** 2021-12-02

**Authors:** Frank Sanner, David Sonntag, Norbert Hambrock, Matthias Zehnder

**Affiliations:** ^1^ Dental Office Frankfurt Germany; ^2^ Carolinum Clinic of Conservative and Preventive Dentistry Frankfurt am Main Germany; ^3^ Dental Office Drachten The Netherlands; ^4^ Clinic of Conservative and Preventive Dentistry Center of Dental Medicine University of Zurich Zurich Switzerland

**Keywords:** endodontic diagnostics, International Classification of Orofacial Pain, persistent idiopathic dentoalveolar pain

## Abstract

**Aim:**

To assess whether persistent idiopathic dentoalveolar pain (PIDAP), a diagnosis of exclusion, exhibits common features that can facilitate its diagnosis. PIDAP is defined by the International Classification of Orofacial Pain (ICOP 6.3.) as ‘Persistent unilateral intraoral dentoalveolar pain, rarely occurring in multiple sites, with variable features but recurring daily for more than 2 h per day for more than 3 months, in the absence of any preceding causative event’.

**Methodology:**

In this observational study, participants fulfilling the new ICOP diagnostic criteria of PIDAP were included, covering 16 years of consecutive data. A systematic, retrospective assessment of patients utilizing the new PIDAP criteria on complaints of chronic tooth pain in a referral‐based endodontic practice was undertaken. Non‐idiopathic cases were excluded on the basis of clinical and radiologic findings. A modified neuropathic pain questionnaire was used to describe the painful sensations. Furthermore, allodynia in the periodontal space and sensory changes in the oral mucosa were assessed using a periodontal probe.

**Results:**

Amongst the 160 patients assessed, 78 (63 women) fulfilled the strict PIDAP criteria. Pain history of PIDAP included no nocturnal awakening (85%) and a ‘pulling/dragging’ pain quality (59%). In 69% of the patients with PIDAP, pain was associated with a root filled tooth at the same site. In 14% of the cases, no endodontic treatment was performed in the affected quadrant. Mechanical allodynia in the gingival sulcus was observed in 91% of patients with painful teeth or implants.

**Conclusions:**

In this observational study, PIDAP mainly affected females and was associated with undisturbed sleep and periodontal allodynia.

## INTRODUCTION

Dental pain is a relatively common condition with a significant socioeconomic impact (Constante et al., 2012). The main reason for this pain is usually endodontic infection (Zehnder & Belibasakis, 2015). However, whilst toothaches from endodontically involved, i.e. infected teeth can be excruciatingly intense, they do not normally last long before the patient seeks dental care (Rechenberg et al., 2016). This form of ‘typical’ tooth pain usually resolves after endodontic intervention (Law et al., 2015). On the other hand, patients with persistent toothache require dentists to differentiate between odontogenic, non‐odontogenic and mixed pain. In contrast to ‘typical’ tooth pain, pain with a neuropathic or idiopathic character presents a greater clinical challenge. The pathophysiology (Vickers & Cousins, 2000), classification (ICOP, 2020), risk factors (Aggarwal et al., 2010), diagnosis, prognosis and therapy for persistent dentoalveolar pain have been discussed extensively (Malacarne et al., 2018). However, despite newer classifications being published, many open questions remain.

The new International Classification of Orofacial Pain (ICOP) classification emphasizes the difference between neuropathic and idiopathic pain that can result in persistent dentoalveolar pain. The latter was the subject of this observational study. In this context, ‘Idiopathic’ is defined as ‘arising spontaneously or from an obscure or unknown cause’ (Merriam‐Webster 2021 https://www.merriam‐webster.com/dictionary/idiopathic).

Patients experiencing persistent dentoalveolar pain experience significant psychological strain that adversely affects their quality of life (Shueb et al., 2015). Persistent idiopathic dentoalveolar pain (PIDAP), which has been redefined most recently (ICOP, 2020), is mainly a diagnosis of exclusion. For the clinician, however, the typical features of PIDAP can help to better distinguish this type of pain from odontogenic and other possible pain conditions at an early stage.

Thus, the goal of this observational study was to describe patients with PIDAP from the dentist's perspective and to identify possible common features within this cohort. From a series of consecutive patients complaining of chronic tooth pain in a referral‐based endodontic practice, patients who fulfilled the ICOP diagnosis of PIDAP (ICOP, 2020) were included over a period of 16 years. The focus of this observational study was on pain history, dental signs and symptoms.

The data collected in this study made it possible to apply the new ICOP criteria for PIDAP and rule out other possible causes for chronic or persistent pain to the maximum extent by characterizing this group of patients.

## MATERIALS AND METHODS

### Patient evaluations

**FIGURE 1:**
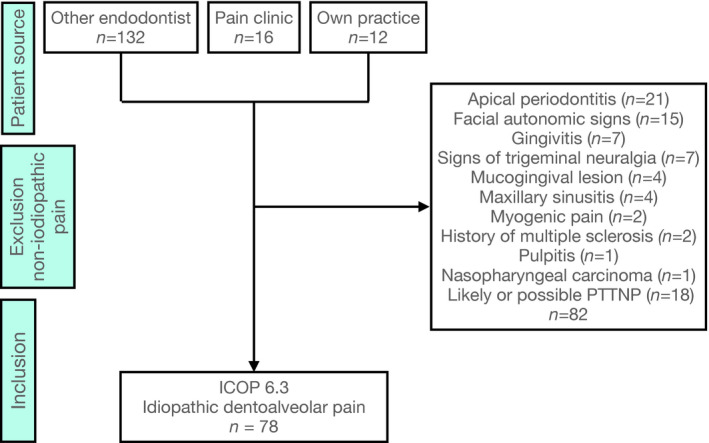
Patient flow classifying patients with persistent idiopathic dentoalveolar pain (PIDAP) according to the International Classification of Orofacial Pain (ICOP)

Prospective data collection in all patients with chronic tooth pain was performed by the principal investigator (F.S.), a trained endodontist and a physician with a focussed interest in pain, between January 2003 and June 2019. The use of this censored data for publication was approved by the local ethics commission (LZK Hessen, 01/2020). The majority of patients were referred from other endodontists within Germany because of ongoing pain after treatment (Figure 1). Patients aged >18 years who had experienced pain persisting for more than three months at the time of the examination were eligible for inclusion. All 160 patients reported pain related to a tooth or extraction site.

Good‐quality periapical radiographs of the painful regions were available and examined in all patients. Any signs of caries, fractures, bleeding on probing, pathological pocket depths (>3.5 mm), mucogingival lesions, internal or external resorption or possible lesions of endodontic origin resulted in the exclusion of the patient from the study group (Table 1). Patients suspected to have pain of a dental inflammatory or traumatic nature or due to other sources of pain related to teeth were excluded from this analysis, as were patients with suspected pain referred from adjacent anatomic structures, such as acute sinusitis, sialadenitis, myofascial pain, arthrogenic pain and signs of trigeminal neuralgia (Figure 1). Patients with a history of external trauma and iatrogenic injuries from dental treatments such as local anaesthetic injections, extractions, root canal treatment, oral surgery, dental implants, orthognathic surgery and other invasive procedures in the painful area within 6 months before the onset of pain were also excluded because of possible or likely post‐traumatic trigeminal neuropathic pain (PTTNP) according to ICOP 4.1.2.3 (ICOP, 2020) when sensory changes in a neuroanatomically plausible area were observed.

**TABLE 1:** Exclusion criteria based on pain type

Relevant clinical findings and case history	Diagnosis
Vertical knocking pain, apical tenderness, evidence of deep caries, mobile restorations, internal or external resorption, crown or root fractures of the affected teeth or their neighbours or antagonists, pain to heat or cold when applied to the teeth.	Odontogenic pain: Symptomatic pulpitis, Symptomatic apical periodontitis.
Bleeding on probing, pathological pocket depths, mucogingival lesions.	Periodontal and mucogingival pain.
Myogenic trigger points or active or passive movements that reproduce the typical pain, radiographic signs of maxillary sinusitis, pain in the tuber region or paranasal pressure pain, swollen and sensitive salivary glands, medical history or clinical examination findings relevant to head and neck tumours or sickle‐cell anaemia.	Referred orofacial pain, head and neck tumours, sickle‐cell anaemia.
Symptoms of the autonomic orofacial nervous system (e.g. running tears, running nose) associated with the intensity of the pain. Occurrence of other typical constellations of findings listed in the ICOP 5.1–5.3 definition (International Classification of Orofacial Pain, ICOP, 2020) or pain relief by triptans.	Various incarnations of facial headache pain.
Intense shock‐like pain within the affected trigeminal distribution and occurrence of typical general medical and specific pain‐related history as listed in ICOP 4.1.1 (ICOP, 2020).	Trigeminal neuralgia.
A neurological disorder known to be capable of causing and explaining, the trigeminal neuropathic pain has been diagnosed and occurrence of diagnostic criteria as listed in ICOP 4.1.2.4 (ICOP, 2020).	Trigeminal neuropathic pain attributed to other disorders.
Pain in a neuroanatomically plausible area within the distribution(s) of one or both trigeminal nerve(s) and a history of trauma to the peripheral trigeminal nerve(s) and other criteria listed in ICOP 4.1.2.3 and 4.1.2.3.1 (ICOP, 2020). Criterion C: History of external trauma and iatrogenic injuries from dental treatments such as local anaesthetic injections, root canal therapies, extractions, oral surgery, dental implants, orthognathic surgery and other invasive procedures within 6 months before the onset of pain Criterion D: Associated with somatosensory symptoms and/or signs in the same neuroanatomically plausible distribution.	Possible or likely post‐traumatic trigeminal neuropathic pain (PTTNP).

Note

Only the most relevant criteria are described in this table (see text).

All pain interviews and clinical examinations were conducted by the principal investigator. After their dental examinations reported here, the patients diagnosed with PIDAP were referred to headache and facial pain clinics or specialized pain therapists.

### Medical history

The medical history was obtained using a health questionnaire that included questions pertaining to current health status (Appendix S1) and the use of prescribed drugs taken on a regular regimen, based on the answers given by the patients.

### Pain questionnaire and interview

All patients completed a pain questionnaire for atypical or persistent tooth pain. This questionnaire (Appendix S2) consisted of 46 standard questions, first assessing the main location of the pain and then addressing the onset, intensity, quality, medications, sleep patterns and other possible contributing factors. A history of surgery or trauma to the head and neck, history of root canal treatments and diseases of the nervous system were investigated. In patients who underwent root canal treatment in teeth associated with the painful site, the sequence of events was noted. This questionnaire was based on a standard neuropathic pain questionnaire (Krause & Backonia, 2003) and recommendations made by Goulet (2001). Additionally, pain‐related symptoms of the orofacial autonomic system and symptoms related to other orofacial structures were addressed. The patients then reviewed their questionnaires with their examiners, who obtained clarification for ambiguous answers. The 11‐point numeric pain rating scale (NRS‐11) was used to rate pain intensity (Warren Grant Magnusson Clinical Center, 2003).

### Clinical examination

The area or tooth producing the most pain, as indicated by the patients, was recorded. All patients underwent the same intra‐ and extraoral examinations. Basic clinical examinations were performed as described in standard endodontic texts (Berman & Hartwell, 2006). Teeth were examined for root fractures and caries with ×4.3 dental loupes (Zeiss). When a root fracture was suspected, the coronal restorations were removed for better visualization.

Accessible masticatory muscles and accessible parts of both TMJs were palpated, and active and passive jaw movements were performed. The patient was asked whether their typical pain could be reproduced during the examination, and if so, they were excluded from the study.

The periodontium was tested for bleeding on probing or pathological pocket depths (>3.5 mm) circumferentially. Patients presenting with one or more of these symptoms in the painful region were excluded from this study (Figure 1). All teeth were tested for mechanical allodynia of the gingival sulcus (‘pain induced by stimuli that are not normally painful’; International Association for the Study of Pain https://www.iasp‐pain.org/resources/terminology/#allodynia) using a periodontal probe (Periodontometer PCP 12; Hu‐Friedy) and applying light pressure. Patients were not asked before probing whether they had experienced any pain. Patients who had a reaction indicating that they might have felt pain were asked to state explicitly whether or not the touch was painful. The reaction was then compared with the corresponding reaction to the same procedure on the contralateral side. The original painful procedure was then repeated, and the patient was asked to comment on any differences compared with the non‐painful contralateral side. Probing of the gingival sulcus was performed in all quadrants.

The attached gingiva and mucosa in the painful quadrant were tested by touching them lightly with a periodontal probe and asking the patient for comparison to the contralateral side. The patient's reactions were also noted. No sensation, less sensation, painful sensations, or an unpleasant sensation in the painful quadrant compared with the contralateral side were classified as numbness, hypoesthesia or allodynia according to the IASP terminology (https://www.iasp‐pain.org/resources/terminology/) or as an unpleasant sensation (dysesthesia). If at least one of these symptoms was present, it was noted as a sensory change in the gingiva in the painful quadrant.

### Statistical analysis

The RStudio software tools (https://rstudio.com/products/rstudio/download/) were used to perform a descriptive statistical analysis. Normally distributed data sets (Shapiro–Wilk test) are presented as means and standard deviations, whilst non‐parametric data are presented as medians and ranges.

## RESULTS

### Patients considered for inclusion

In total, 160 patients with persistent dental pain were assessed. Sixty‐four of these were excluded since odontogenic pain, facial headache variants, referred pain and other non‐idiopathic pain sources could not be ruled out or signs of gingivitis were present (Figure 1). Eighteen patients with persistent pain were excluded because of the criteria for PTTNP. Two of them had injection injuries to the inferior alveolar nerve, 10 had undergone root canal treatments within 6 months and sensory changes were noted in a neuroanatomically plausible area. Six patients with a history of root canal treatment within 6 months and no apparent sensory changes were excluded from this study because not all possible sensory changes could be ruled out (see Appendix S3)

None of the patients were pregnant or had a history of herpes zoster infection in the head and neck area.

#### PIDAP patients

Seventy‐eight patients (63 women, 15 men) met the inclusion criteria for this study and were diagnosed with PIDAP. The site of pain was the teeth of 75 patients, implants in one patient or edentulous post‐extraction sites in two patients (Table 2). The painful jaw was the mandible in 33 patients and the maxilla in 45 patients. The region of the first molars was the most frequently reported painful site (32%). In 54 of the 78 patients, the painful site had been endodontically treated prior to examination. In all cases with painful implant‐ or toothless post‐extraction sites, the preceding extraction was associated with a formerly root canal‐treated tooth. In 24 cases, the painful tooth had no clinical or radiological signs of pulp necrosis and exhibited a normal reaction when applying the cold test. Of these 24 patients, 13 had undergone root canal treatment in the same quadrant as the painful tooth. In only 14% of the cases, root canal treatment was performed neither in the painful tooth nor in the quadrant.

**TABLE 2:** PIDAP patient characteristics, pain duration, intensity and location

Sex (female/male)	63/15
Age in years (mean ± SD)	49 ± 14
Pain duration in years (median/IQR)	1.5/(1.4)
Mean NRS‐11 pain intensity during the last 2 weeks (median/IQR)	3/(2.5)
Maximum NRS‐11 pain intensity during the last 2 weeks (median/IQR)	8/(5.9)
Affected jaw (maxilla/mandible)	45/33
Tooth region (molar/premolar/front)	38/25/15
Dentition at the painful site (tooth/edentulous/implant)	75/2/1
History of root canal treatment at the painful site (yes/no)	54/24

Abbreviations: IQR, inter‐quartile range (0.25 Quartile, 0.75 Quartile); NRS, Numerical Rating Scale for pain intensity; SD, standard deviation.

In 25 cases in the PIDAP group, the persistent pain was preceded for more than 6 months by root canal treatments or other dental procedures that are listed to be relevant to a diagnosis of PTTNP in the ICOP classification, such as extractions, oral surgery, dental implants, orthognathic surgery and other invasive procedures. In 12 cases, persistent pain preceded the root canal treatment, and in 24 cases, no root canal treatment or other invasive dental procedures were performed on the tooth. In 12 cases, the root canal treatment preceded the beginning of persistent pain for less than 6 months, but since sensory changes were found in other quadrants as well, these cases met the criteria for PIDAP, not PTTNP (Appendix S3). Cases in which the timing of dental procedures was not known were not included in the study.

### Medical history and medication usage

Other chronic pain conditions reported by patients with PIDAP included chronic back pain (8 individuals), irritable bowel syndrome (3), migraine (4), fibromyalgia (2) and tension headache (1). A history of depression was reported in nine cases and psychosis in one. The most frequently reported medical conditions were thyroid disorders (21), including 12 cases of Hashimoto's thyroiditis. Eleven patients had cardiovascular and respiratory disorders. Other disorders or diseases were present in very small numbers of patients. Twenty‐one patients reported no other health problems besides the existing persistent intraoral pain.

Drugs taken on a regular basis included l‐thyroxin (18), contraceptive medication (4), drugs used for chronic pain treatment (8), psychotropic drugs (3) and cardiovascular drugs (10).

### Pain characteristics

The pain had been present for an average period of 2.7 years before the examination (median: 1.5; range: 4 months to 13 years). A group of 17 women had pain persisting for more than or equal to five years. The maximum pain intensity during the 2 weeks prior to assessment ranged from 2 to 10 on the NRS‐11 scale (median: 8; mean: 7.2). The mean average pain score in the same period was 3.8 (median: 3), and the mean NRS‐11 scores for female and male patients were 3.8 and 4.1, respectively.

Sixty‐six of the 78 (85%) patients stated that the pain did not wake them at night. Furthermore, 64 (82%) patients experienced pain‐free intervals on waking in the morning before the pain set in. Pain was present during the daytime for more than 2 h in all patients.

Patients generally used more than one term to describe the nature of their pain. The most frequently used adjectives were as follows: ‘pulling/dragging’, reported by 46 of 78 patients (59%); ‘pressing’ by 41 (52%); ‘burning’ by 33 (42%); ‘tingling’ by seven (9%); ‘electric’ by three (4%); and ‘stabbing’ by 14 (18%). Forty‐three (55%) patients reported experiencing pulsating or throbbing pain at the affected site from time to time. When describing the quality of their pain in more words, patients frequently used non‐standard, figurative expressions that differed from more common ways of describing toothaches, such as ‘like being stabbed with a knife’, ‘like a wound’, ‘like dead tissue that is nevertheless painful’, ‘as if the tooth were to explode’ and ‘as if the bones were moving’.

### Clinical findings

Pressure to the periapical bone exerted by digital palpation was never painful, nor could pain be elicited by vertical tapping on a tooth with an instrument in any case. Discomfort was elicited by horizontal tapping in six patients. Mechanical allodynia of the gingival sulcus was found in 68 of 75 teeth and in one implant. Thus, allodynia in the periodontium/peri‐implant mucosa was observed in 69 of 76 patients with painful teeth or implants (91%). In two of the cases in which the pain affected the edentulous region, this sign could not be tested. The painful section invariably consisted of only part of the gingival sulcus and never included its entire circumference. The same symptoms could frequently be elicited in the sulcus of the adjacent teeth or in the antagonistic or contralateral quadrants. An allodynic area in the gingival sulcus of the neighbouring teeth was present in 53 cases. More than one quadrant was affected in 39 cases: two quadrants in 22 cases, three quadrants in 11 cases and all four quadrants in six cases.

The gingiva and mucosa of all patients were checked for sensitivity changes to touch by using the tip of a periodontal probe to slightly touch the gingiva and mucosa in the painful quadrant, first on the non‐painful contralateral side and then on the affected painful side. In 9 cases, patients reported numbness near the site of the pain after repeated comparisons with the non‐painful contralateral side. None of the patients had been aware of this numbness before the examination. Twenty‐eight patients showed sensitivity changes other than numbness in the painful quadrant beyond the gingival sulcus; mechanical allodynia was present in five patients. Seven patients reported reduced sensitivity to touch (hypoesthesia) and 15 reported discomfort when touched with a periodontal probe compared to the non‐painful side. In seven patients, more than one sensitivity change in the fixed gingiva was present concurrently.

The painful sensation sometimes increased as a result of the examination, especially on contact with the gingival sulcus. A total of 32 patients reported an increase in pain levels after completion of the examination.

## DISCUSSION

In this observational study, strict PIDAP criteria were applied to illuminate the condition from the dentist's perspective. This allowed the identification of PIDAP features that have not been described previously, including the absence of nocturnal pain and the presence of allodynia in otherwise healthy periodontal sites.

### PIDAP patients

Until recently, the nomenclature for the conditions under investigation was not standardized (Nixdorf et al., 2012). A new ICOP was introduced in 2020 (ICOP, 2020), which was followed here. Studies on atypical odontalgia in endodontic patients have reported an occurrence rate of <5% (Campbell et al., 1990). An association between persistent toothaches and endodontically treated teeth has also been reported (Nixdorf et al., 2010).

The association between root canal‐treated teeth and non‐odontogenic tooth pain could be explained by either pathophysiological changes under special circumstances following root canal treatment that led to pain chronification or, more likely, indicating that non‐odontogenic tooth pain was present from the beginning and a root canal treatment was performed with the intent to help the patient. This point should be addressed in future studies. Sex dependence of chronic pain has been frequently described (Mogil, 2020). A previous study reported a predominance of female patients (List et al., 2007), which was also confirmed in this study.

### Medical history

Thyroid disorders (27%), chronic pain conditions (23%), and depression (10%) were reported by the patients. Because this information was questionnaire‐based, its completeness and accuracy were not assured. It was only included to describe the group of patients under investigation in more general terms. Nevertheless, the relatively high frequency of thyroid disorders in this cohort warrants further research. An examination of a large sample of self‐reported chronic pain (McWilliams et al., 2003) found that the incidence of apparent thyroid disease amongst individuals affected by chronic pain was 5.5%.

### Pain characteristics

The maximum pain intensity of the PIDAP patients under investigation that was reported for the two weeks prior to clinical assessment was similar to that of a cohort of endodontic emergency patients with infection‐related pain (median of 8 on the NRS‐11 scale) (Rechenberg et al., 2016). However, and more importantly, the finding that the pain did not wake patients at night in these individuals with PIDAP sets their condition apart from infection‐related tooth pain. In the study by Rechenberg et al. (2016), 80% of emergency patients experiencing acute pulpitis and 83% of those with acute apical periodontitis stated that their pain woke them at night. An increase in intensity when lying down or resting is typical of inflammation‐related pain. Atypical odontalgia that affects patients mostly at and throughout the day, on the other hand, has been reported previously (Rees & Harris, 1979) but has never been specifically assessed.

Similar pain descriptors as in this study have been reported previously in the literature (List et al., 2007). A striking typical pain characteristic in the population studied here is the burning pain quality, which affected 42% of the patients. Burning pain is also generally described as a characteristic of neuropathic pain (Collaca et al., 2017). Undisturbed sleep and a pain‐free interval after waking are typical characteristics of neuropathic pain (Odrcich et al., 2006).

A delayed onset of pain following irritation (41%) has also been described in neuropathic pain. In this regard, mechanisms similar to those of ‘wind‐up’, a progressive, frequency‐dependent increase in excitability, may play a role (Coste et al., 2008).

### Clinical findings

Persistent idiopathic dentoalveolar pain was the most frequent diagnosis amongst patients with chronic dentoalveolar pain. In addition to nociceptive and inflammatory pain, as is the case with odontogenic pain, from a pathophysiological point of view, there are two other pain types frequently implicated in chronic pain, namely neuropathic pain (Finnerup et al., 2016) and neuroplastic pain (Kosek et al., 2016).

The ICOP classification describes the type of pain under investigation as idiopathic.

The diagnosis of non‐odontogenic toothaches is usually a differential diagnosis based on the absence of any explanatory tooth‐related pathology. Although the sign of non‐response to local anaesthesia was not examined in this study, such non‐response at the site of the pain (List et al., 2006) can be helpful in the diagnostic process.

#### Sensory changes

Simple methods available to any general dentist can be applied to detect gross sensory changes in the gingiva and mucosa. Somatosensory tests such as the ‘Quantitative Sensory Test’ (QST) (Maier et al., 2010) and the ‘Qualitative Sensory Test’ (QualST) (Baad‐Hansen et al., 2013) have been described for sensory testing. The present data collection started earlier than the description of the sensory tests described above. Nevertheless, a different aspect of sensory testing has been emphasized. A simpler approach using a periodontal probe was followed. The area connected most closely to the dental nerve and pulp is the periodontium and hence the gingival sulcus. Pulpal and periodontal innervation develop concurrently with the development and eruption of the dentition. Innervation occurs during tooth eruption (Fristad et al., 1994). The periodontal space is characterized by unique innervation. Sensory changes in this region related to PIDAP have not yet been studied, nor do the intraoral variants of QST and QualST cover this aspect. Quantitative sensory testing is mentioned as a means for further specifying PIDAP into 6.3.1 with and 6.3.2 without somatosensory changes. Further discrimination of PIDAP depending on sensory findings must be taken into consideration, since this classification is very dependent on the methods involved. Although extraoral QST has been standardized (Matos et al., 2011), and intraoral QST has been described (Baad‐Hansen et al., 2015), more practical means for dental practitioners are needed and developed in the form of QualST (Baad‐Hansen et al., 2013).

Probing of the gingival sulcus is usually not painful. In the present study, a high percentage of patients (91%) had this sensory abnormality, often not only in the painful tooth but also in other teeth and quadrants. Neurophysiological research on this topic has shown that trauma to one dental nerve may alter the processing of pain in neighbouring teeth and other quadrants (Kondo et al., 1992; Sabino et al., 2002). Sabino et al. (2002) reported a correlation between the invasiveness of dental procedures and neuroplastic changes in the brainstem. The invasiveness of the procedures was correlated with the extent of changes on the same and contralateral sides. In the present study, allodynia in the gingival sulcus, which could be a result of neuroplastic changes, was also found in 68% of neighbouring teeth and in 50% of other quadrants. More insights can be gained into this topic by obtaining better knowledge of the innervation of the junctional and oral epithelium within the sulcus and the periodontal ligament, the sensory changes within the periodontal space, and the changes in the nerve following separation of the pulp from its nerves, as they occur during endodontic treatment.

The inclusion of mechanical allodynia within the gingival sulcus into the already described PIDAP diagnostic features may provide additional important diagnostic utility.

### Strengths, limitations and outlook

The strengths of this study include the fact that an attempt was made to systematically exclude nociceptive odontogenic pain, referred pain, facial headaches and post‐traumatic neuropathic pain to the maximum extent possible. The inclusion and exclusion criteria were chosen to avoid creating a mixture of different characteristics within the group with otherwise unexplained non‐odontogenic pain (headache variants, referred pain, odontogenic pain) and to identify characteristics of specific groups that may be due to changes in the pain processing system typical of PIDAP.

Nevertheless, this observational study was limited by the fact that it was not a cross‐sectional study, and the pain and treatment history depended on the patient's memory. As a result, no conclusions can be drawn regarding the prevalence of the conditions under investigation. Furthermore, there was a selection bias because patients were mostly seen in endodontic clinics (Figure 1), which represents a specific cohort and may not be representative. However, because PIDAP is experienced in teeth, patients frequently seek help from endodontists, or cases are referred because general practitioners have already initiated root canal treatment. Another limitation of this study is that no comparisons were made pertaining to thesigns and symptoms that were identified in the current cohort in comparison to patients experiencing infection‐related tooth pain. Nevertheless, the current results can be compared with reported data in the literature on both atypical odontalgia and in contrast to ‘typical’ infection‐related tooth pain (Rechenberg et al., 2016). Since there is a regional and cultural element to pain (Peacock & Patel, 2008), it has to be taken into consideration, that the study was performed on a German patient population.

The focus of the current work was on how PIDAP patients presented in endodontic practice, and which were the key features of their condition from a dental perspective. In a future study, an attempt will be made to follow the PIDAP patients identified here to assess how their pain was managed.

### Clinical implications

- PIDAP is normally a diagnosis of exclusion, but common features may exist;
- No nocturnal awakening due to pain was reported by most patients with PIDAP;
- The sulcus around the affected tooth/implant was painful upon light touch of the periodontal probe in a significant proportion of PIDAP patients;
- PIDAP mainly affected females;
- The five most frequent pain descriptors were ‘pulling/dragging’ (59%), ‘pulsating’ or ‘throbbing’ (55%), ‘pressing’ (52%) and ‘burning’ (42%).

## CONCLUSIONS

Persistent idiopathic dentoalveolar pain is mainly diagnosed by exclusion. For clinicians, the typical features of PIDAP can help to better differentiate this type of pain from odontogenic and other possible pain conditions. The identification of allodynia in the gingival sulcus of affected teeth/implants appears to be a common PIDAP feature easily detected by the dentist, as is the information that the pain does not wake the patient at night. In view of the rarity of PIDAP, special networks or centres are needed to generate a sufficient number of cases whose evaluation can provide the basis for an improvement in the diagnosis and treatment of patients with persistent toothaches.

## ETHICS STATEMENT

The use of data pertaining to this observational study was approved by the local ethics committee (LZK Hessen, 01/2020).

## CONFLICT OF INTEREST

The authors have stated explicitly that there are no conflicts of interest in connection with this article.

## Supporting information

Appendix S1

Appendix S2

Appendix S3

## Data Availability

Frank Sanner: clinical assessments, data collection, data curation, analysis, and writing. David Sonntag: conceptualisation and writing. Norbert Hombrock: data collection and review of manuscript. Matthias Zehnder: conceptualisation, data curation, analysis, and writing.
