# Supplementary material for: Patients with persistent idiopathic dentoalveolar pain in dental practice
Source: Int Endod J. 2021 Dec 2;55(3):231–9. doi: 10.1111/iej.13664 (PMC9300172; doi:10.1111/iej.13664)
Supplement: Supplementary file 1 — Appendix S1 [file IEJ-55-231-s001.docx]

QUESTIONS REGARDING HEALTH STATUS AND MEDICATIONS

(Questions relevant to the study out of the standard questionnaire regarding health and oral problems every patient filled out after entering the dental office)

Do you suffer from dental or gingival pain?

Are your teeth sensitive to heat or cold?

Did you notice halitosis?

Do you suffer from gum bleeding?

Did you have a periodontal treatment performed by your dentist?

Are you happy with the status of your teeth or dental restaurations?

Do you clench or press your teeth at night?

Did you experience clicking or other noises in front of the ears when you move your jaws?

Is your jaw movement painful?

Did you experience restricted movements when you move your jaws?

Do you smoke?

Have you been diagnosed or do you know of any of the following diseases or disorders:

Diabetes

Thyroid disease

Pulmonary disease/Asthma

Liver disease/jaundice

Rheumatic disease/rheumatic fever

Gastrointestinal diseases

Kidney problems

Seizures

Allergies (which?)

Diseases of the nervous system

Problems with blood clotting

Immune deficiency (HIV)

Neoplastic disease

Glaucoma

High blood pressure

Low blood pressure

Cardiac disease (pacemaker, Hx of cardiac surgery, valve disease, Hx of endocarditis, prosthesis of cardia valve)

Do to take drugs affecting blood clotting (eg Markumar ®)

Are there any known drug intolerances?

Which drug are you currently taking: ______________

Are there any other health problems or diseases not mentioned above?
