# Supplementary material for: Patients with persistent idiopathic dentoalveolar pain in dental practice
Source: Int Endod J. 2021 Dec 2;55(3):231–9. doi: 10.1111/iej.13664 (PMC9300172; doi:10.1111/iej.13664)
Supplement: Supplementary file 2 — Appendix S2 [file IEJ-55-231-s003.pdf]

Questionnaire

for orofacial pain patients

Date:

Attending dentist in this office:

Patients name and age:

Names of medical and dental colleagues currently involved:

Description of current pain problem:

1. a) Please mark the location of maximum pain and if it radiates in any direction

In case of more than one simultaneous pain location number and mark the other locations

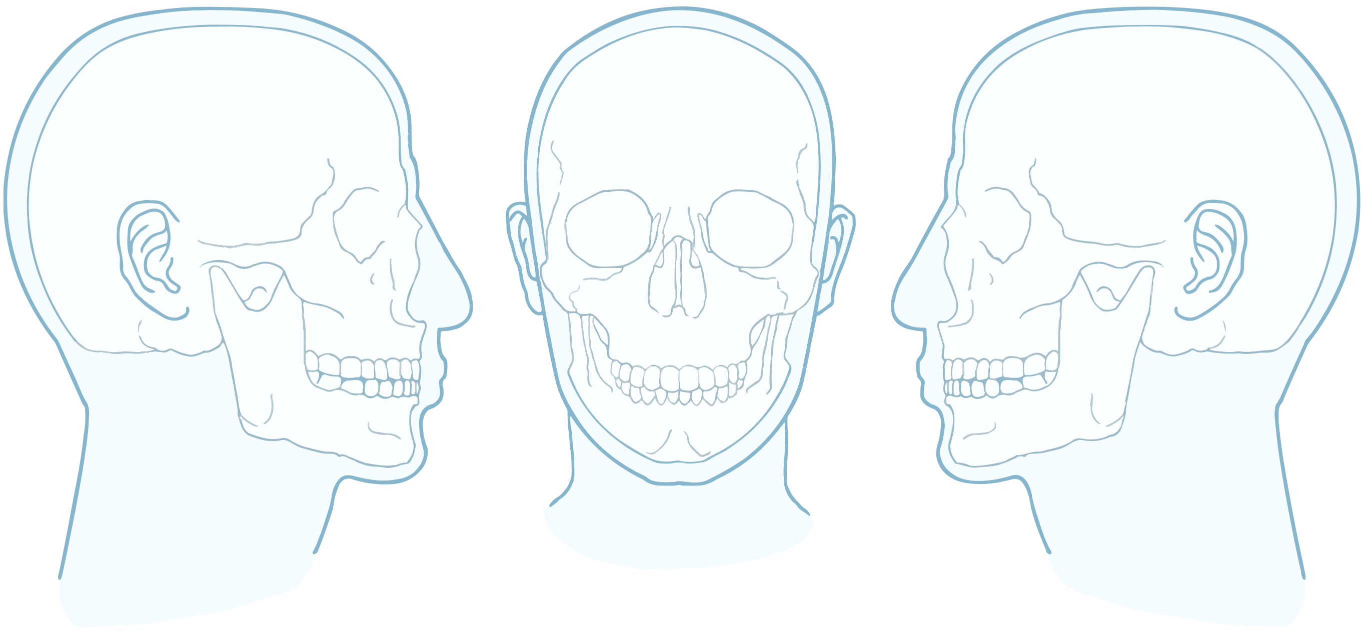

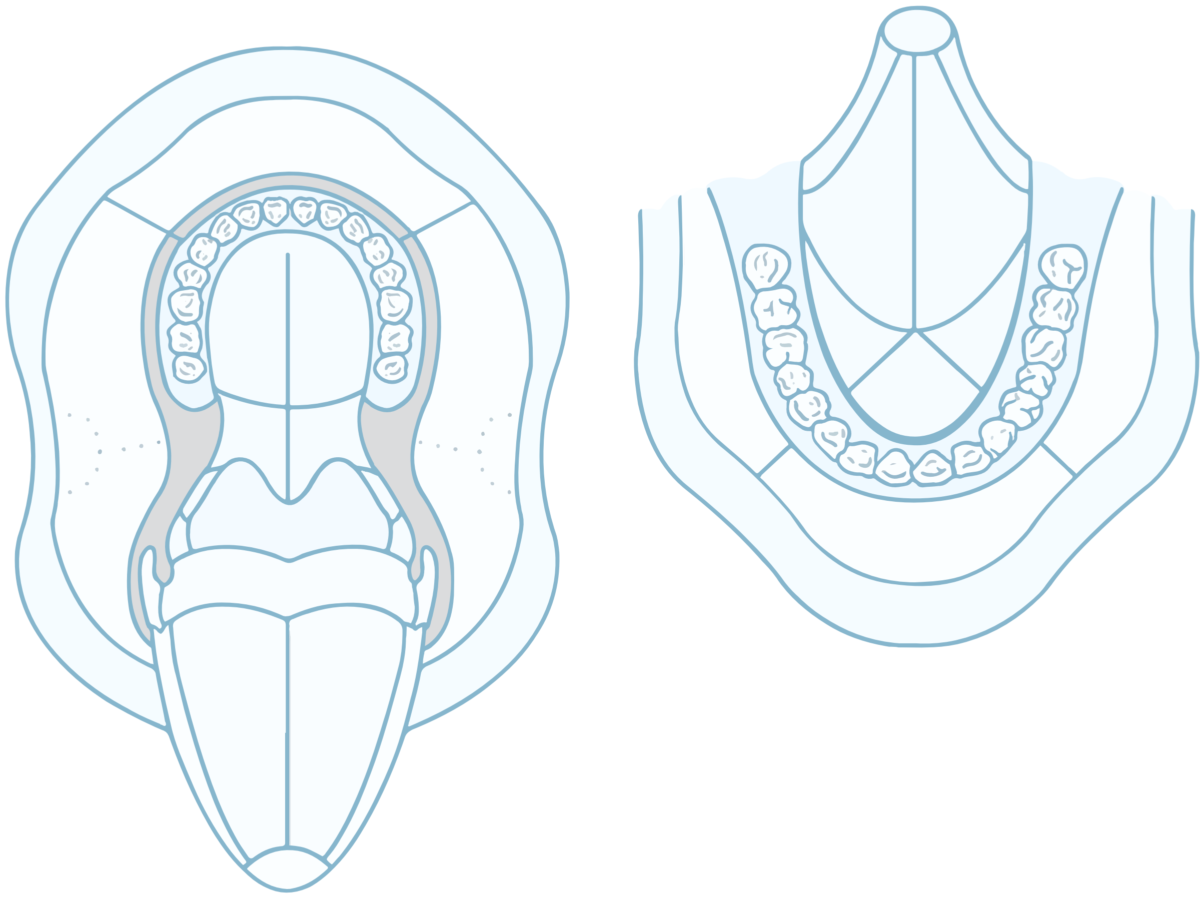

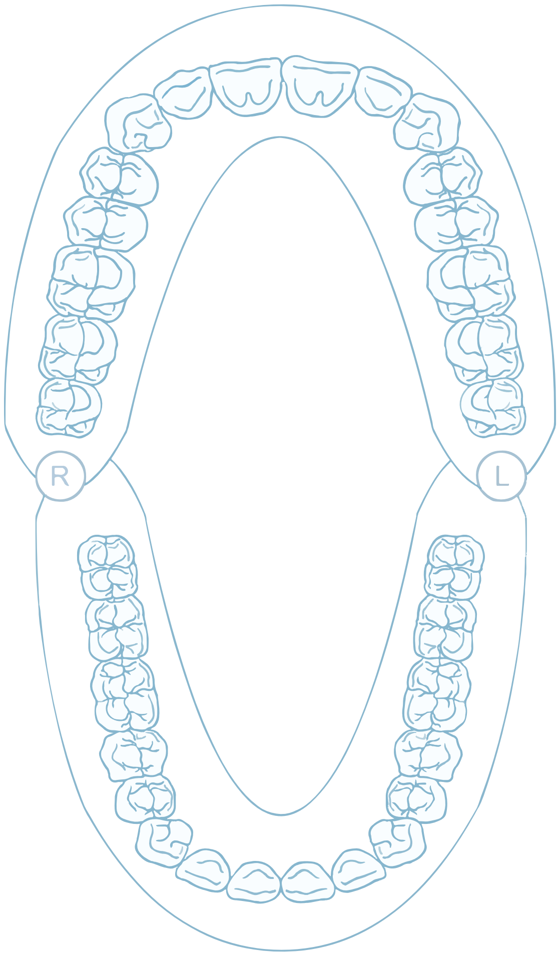

b) Please mark other pain locations if present:

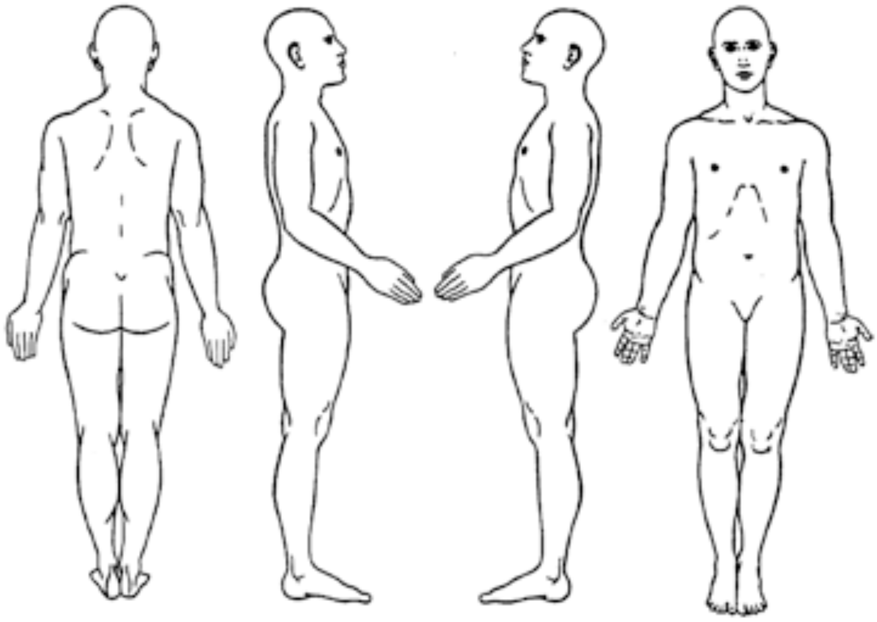

Timeline of events since first appearance of pain:

2. a) What number would you give your pain right now, if 0 means no pain and 10 means worst possible pain?

no pain	0	1	2	3	4	5	6	7	8	9	10	worst possible pain

b) What number would you give your average pain during the past 4 weeks?

0	1	2	3	4	5	6	7	8	9	10

c) What number would you give the maximum pain during the past 4 weeks?

0	1	2	3	4	5	6	7	8	9	10

3. How would you describe the course of pain over time?

	yes	no	don't know
a) Continuous pain with slight undulations?	☐	☐	☐
b) Continuous pain with explicit undulations?	☐	☐	☐
c) Bouts of pain, with no pain in between?	☐	☐	☐
d) Bouts of pain, with pain in between?	☐	☐	☐
e) Are there times when the pain is completely gone?	☐	☐	☐

f) In case of bouts of pain: how long do they last? ______________________________

e) How often do you have episodes of increased pain (on a daily/ monthly basis)? __________ / __________

	yes	no	don't know
g) Does the pain sometimes start like a flash?	☐	☐	☐

h) Could you please mark the changes in pain level during one typical day?

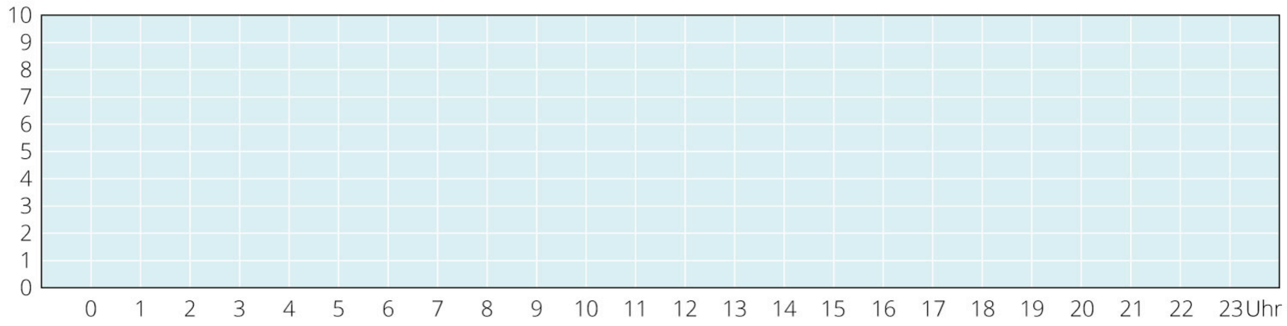

4. When did you first experience the pain?

5. Was this connected to a special event?

	yes	no	don't know
6. Does the pain change during the course of the day?	☐	☐	☐

7. Which diagnostic and therapeutic measures were taken and did the pain change following the therapeutic measures?

8. Did the pain change during it started in regard to the location, quality and intensity?	☐	☐	☐
9. Does the painful tooth fell „higher“ than usual?	☐	☐	☐
10. Did the pain wake you from sleeping?	☐	☐	☐
11. Is the pain present directly after waking up or did you notice a pain free interval after waking up?	☐	☐	☐

12. Is the pain strictly limited to one side or does it alternate?

13. How would you describe the pain („burning“, „aching“, „throbbing“, …) ?

14. Did you experience trauma or surgical interventions in the painful area?	☐	☐	☐

15. What makes the pain worse (exercise, neck movement, chewing, dinking alcohol, temperature changes, …)?

16. Is there anything that can make the pain go away or soothe the pain?

No.	Question	yes	no	don't know
17.	Did you still experience the pain while the area was numb due a dental local anesthesia that was administered to this area?	☐	☐	☐
18.	Did you experience a „root canal treatment“ in the painful area?	☐	☐	☐
19.	Did you experience a sensitivity to cold or hot in the painful area?	☐	☐	☐
20.	Are teeth sensitiv to touch in the painful area?	☐	☐	☐
21.	Is the gum sensitiv to touch in the painful area or does it f wounded?	☐	☐	☐
22.	Does the gum feel numb in the painful area?	☐	☐	☐
23.	Is there a burning sensation in the painful area?	☐	☐	☐
24.	Did you notice one of the following with the painful episodes?			
	A feeling like „sand in the eye“	☐	☐	☐
	Tearing of the eyes	☐	☐	☐
	Running nose	☐	☐	☐
	Stuffy nose	☐	☐	☐
	Hanging eye lid	☐	☐	☐
	Narrowed pupil of the eye	☐	☐	☐
25.	Is there any known problem with the nerve system (e.g Lyme disease, migraine, stroke, …)	☐	☐	☐
26.	Do you experience pain or sensitivity changes in other parts of the body?	☐	☐	☐
27.	Did you experience cold sores or skin alterations in the painful area?	☐	☐	☐
28.	Are you sensitiv to noise, smell or light during the painful episodes?	☐	☐	☐
	If so, is it on the same side as the pain or on both sides			
29.	Do you feel nauseated in connection with the pain level?	☐	☐	☐
30.	Do you press or grind your teeth at night?	☐	☐	☐

31. Are there diseases known of? ______________________

____

____

____

	yes	no	Don´t know
32. Does the pain level increase during exercise ?	☐	☐	☐

33. Which drugs do or did you use for pain treatment and what was their effect?

Name of the drug	Effect

34. Which other measures were used for pain treatment and what was their effect?

Measure	Effect

	yes	no	Don´t know
35. Did you receive bisphosphonates?	☐	☐	☐
36. Does the pain improve when lying down?	☐	☐	☐
37. Does the pain get worse when lying down?	☐	☐	☐
38. Did you have an urge to move when you are in pain?	☐	☐	☐
39. Did you experience an itchy sensation in the painful area?	☐	☐	☐

		yes	no	Donut know
40.	Did you experience a stinging pain sensation?			
41.	Is the pain pressing?			
42.	Does the pain have a dull quality?			
43.	Do you often have a dry mouth?			
44.	Do you awake rested?			
45.	Did you notice a bitter taste or did your sense of taste change?			
46.	Do you experience tinnitus?			

Do you agree with us, that we take pictures or videos during examination?

Yes No

Do you confirm that we are allowed to use your anynomized data from the examination for d nach scientific purposes according to the laws and rules that have to be applied?

Yes No

Thank you for answering the questions!
