# Supplementary material for: Patients with persistent idiopathic dentoalveolar pain in dental practice
Source: Int Endod J. 2021 Dec 2;55(3):231–9. doi: 10.1111/iej.13664 (PMC9300172; doi:10.1111/iej.13664)
Supplement: Supplementary file 3 — Appendix S3 [file IEJ-55-231-s002.pdf]

**Criterion C** Onset of pain in relationship to injury, RCT or other IDP in specified area	**Criterion D** Presence and distribution of neurosensory changes in neuroanatomically plausible area			
Patients with onset of pain immediately or within 6 month: 30	Neurosensory changes in plausible area: 12[1]	→	Possible or likely PTTNP: 12	Exclusion
	Neurosensory changes in plausible area could not be excluded: 6[2]	→	PTTNP can not be ruled out: 6	
	Neurosensory changes not in plausible area: 12[3]	→	PIDAP: 78	Inclusion
Onset of pain 6 month or more later: 25	→			
Prior onset of pain: 17	→			
No history of injury or IDP: 24	→			

SUPPLEMENT 3: Application of PTTNP criteria to 96 patients with persistent dentoalveolar pain after all other exclusion criteria mentioned in table 1 were applied. In cases with onset of patient reported pain immediately or within 6 months after a traumatic event, injury and/or IDP to the specified area (criterion C), criterion D defines the classification, based on the presence of neurosensory changes in a neuroanatomically plausible area. Criteria for PTTNP are listed in ICOP 4.1.2.3 and 4.1.2.3.1 (IHS 2020). Six patients with persistent pain and a history of root canal treatment within 6 months were excluded because the classification criteria could not be applied because not all possible sensory changes could be ruled out.

1: Sensory changes confined to one quadrant 2: No sensory chances found 3: Sensory changes in 2,3 or 4 quadrants.

RCT=„Root canal treatment“, IDP = „Invasive Dental Procedure“ such as RCT, placement of dental implant, tooth extraction.
